# Simulation-based evaluation of operating room management policies

**DOI:** 10.1186/s12913-021-06234-5

**Published:** 2021-03-24

**Authors:** Jan Schoenfelder, Sebastian Kohl, Manuel Glaser, Sebastian McRae, Jens O. Brunner, Thomas Koperna

**Affiliations:** 1 Chair of Health Care Operations/Health Information Management, Faculty of Business and Economics, University of Augsburg, Universitätsstraße 16, 86159 Augsburg, Germany; 2 University Center of Health Sciences at Klinikum Augsburg (UNIKA-T), Neusässer Straße 47, 86156 Augsburg, Germany; 3 Associate Professor of Surgery, Head OR-Management, University Hospital Augsburg, Stenglinstraße 2, 86156 Augsburg, Germany

**Keywords:** Operating room management, Patient scheduling, Patient flow, Capacity management, Simulation

## Abstract

**Background:**

Since operating rooms are a major bottleneck resource and an important revenue driver in hospitals, it is important to use these resources efficiently. Studies estimate that between 60 and 70% of hospital admissions are due to surgeries. Furthermore, staffing cannot be changed daily to respond to changing demands. The resulting high complexity in operating room management necessitates perpetual process evaluation and the use of decision support tools. In this study, we evaluate several management policies and their consequences for the operating theater of the University Hospital Augsburg.

**Methods:**

Based on a data set with 12,946 surgeries, we evaluate management policies such as parallel induction of anesthesia with varying levels of staff support, the use of a dedicated emergency room, extending operating room hours reserved as buffer capacity, and different elective patient sequencing policies. We develop a detailed simulation model that serves to capture the process flow in the entire operating theater: scheduling surgeries from a dynamically managed waiting list, handling various types of schedule disruptions, rescheduling and prioritizing postponed and deferred surgeries, and reallocating operating room capacity. The system performance is measured by indicators such as patient waiting time, idle time, staff overtime, and the number of deferred surgeries.

**Results:**

We identify significant trade-offs between expected waiting times for different patient urgency categories when operating rooms are opened longer to serve as end-of-day buffers. The introduction of parallel induction of anesthesia allows for additional patients to be scheduled and operated on during regular hours. However, this comes with a higher number of expected deferrals, which can be partially mitigated by employing additional anesthesia teams. Changes to the sequencing of elective patients according to their expected surgery duration cause expectable outcomes for a multitude of performance indicators.

**Conclusions:**

Our simulation-based approach allows operating theater managers to test a multitude of potential changes in operating room management without disrupting the ongoing workflow. The close collaboration between management and researchers in the design of the simulation framework and the data analysis has yielded immediate benefits for the scheduling policies and data collection efforts at our practice partner.

**Supplementary Information:**

The online version contains supplementary material available at 10.1186/s12913-021-06234-5.

## Background

Providing appropriate healthcare will be one of the biggest issues for many countries in the upcoming decades. The growth of the population and the impact of an aging population imply an increasing need for healthcare services. Hospitals additionally face increasing pressure to work economically viable and to become more effective. Finally, the supply side is increasingly struggling with meeting the demand, as the already insufficient nurse staffing shows [1]. Since operating rooms are a major bottleneck resource and an important revenue driver in hospitals, it is important to use these resources efficiently. Studies estimate that between 60 and 70% of hospital admissions are due to surgeries [2]. Moreover, around 40% of hospital expenses, as well as revenues, are generated in the operating theaters [3]. Furthermore, the impact on downstream resources such as intensive care or regular ward beds is highly influenced by the surgical program. The necessity of elaborate scheduling is raised by the high uncertainty that comes along with surgeries [4] and the high fixed costs of operating theaters. Furthermore, staffing levels are virtually impossible to adapt to changing demand [5]. The resulting high complexity in surgery scheduling necessitates perpetual process evaluation and the use of managerial decision support tools.

We present a detailed simulation model that is capable of evaluating a variety of managerial policies in a realistic setting. We explore the quantitative effects of a) employing parallel induction of anesthesia in the central operating theatre, b) alternative scheduling policies, c) the increase of operating room (OR) buffer capacity, and d) opening or closing an operating room reserved for emergencies. The analysis is based on a large data set with 12,946 surgery procedures from the University Hospital Augsburg, which is one of the largest German hospitals with over 1700 beds. The hospital is a maximum care provider and therefore has a large variety of highly specialized medical departments to fulfill this public service mandate. The study was performed after the integration of a department from a remote site into the operating theater and before the introduction of new administration software for the management of surgeries.

There is a broad range of academic literature on operating room planning. The extensive amount of literature in the field is both an indicator of the relevance as well as the complexity of operating room planning. We refer to Cardoen et al. [6], Guerriero and Guido [2], and Van Riet and Demeulemeester [7] for overviews on the current state of the literature on operating room planning. In the most recent literature review, Samudra et al. [8] perform an exhaustive search, identify 216 technically oriented papers, and classify these papers according to descriptive fields such as solution techniques. Discrete-event simulation plays a significant role in the following publications to assess the effect of different management policies on various performance criteria, such as utilization or waiting time.

Persson and Persson [9] use discrete-event simulation to study management policy changes in a single operating room department. They employ optimization techniques to identify cost-optimal patient schedules. Adan et al. [10] illustrate the trade-off between hospital efficiency and patient service levels. They use discrete-event simulation to explore the effect of different strategies such as overplanning and flexibility and cancellation policies. Here, elective patients may be canceled to account for emergencies, and emergency patients can be redirected to other hospitals. Berg et al. [11] use simulation to compare booking, sequencing, and scheduling decisions based on a stochastic single server booking model with those employed in practice. Different scenarios, based on varying overtime and fixed costs are evaluated. Azari-Rad et al. find a reduction in the cancellation rate when elective patients with shorter average lengths of stay are scheduled earlier in the week [12]. Furthermore, surgeries with shorter durations and less variance contribute to a reduction of cancellations if scheduled earlier in the day. In addition, the impact of changing capacity in post-surgical ward beds is analyzed. On the other hand, Bowers and Mould show that the throughput may increase significantly if patients are willing to accept the possibility of their treatment being canceled [13]. Finally, Vanberkel and Blake detect that the available operating room time is not the bottleneck of their system in a simulation study [14]. Instead, the available bed capacities are the limiting parameters that interfere with shorter waiting times for patients. The literature review on discrete event simulation in healthcare by Zhan shows that operating theaters have not been the subject of many research efforts that employ simulation modeling as the primary analysis tool, despite both “individual heterogeneity” and “individual interaction” being very much present in this setting [15]. The presence of both properties means that the operating theater setting lends itself well to simulation modeling studies, as other modeling techniques such as Markov models and decision trees typically fail to incorporate either.

Our work extends the literature by addressing additional management policies, by modeling intra- and interday effects of disruptions on schedule execution and rescheduling within the simulation model environment, and by the size and number of the different medical departments in the operating theater. The latter opens up possibilities to introduce shared emergency capacities, patient reallocations, and overtime balancing, among others, to the simulation model.

## Methods

The simulation study presented here originates from a project that spanned over more than a year in close collaboration between the operating theater management and our team of researchers. It involved in-depth process and data analyses, the iterative creation of the detailed simulation environment over multiple feedback loops, and a simulation-based investigation of several potential measures considered by the operating theater management. Note that additional information on the simulation design is available in the Additional file 1. The goal of the simulation study is to evaluate the performance of the following possible management policies per request of the operating theater management at University Hospital Augsburg:

1. What are the minimum staffing requirements for well-functioning overlapping anesthesia?
2. What trade-offs occur when the daily schedule of surgeries is ordered according to the expected surgery duration?
3. Should extended operating hours, and to what extent, be considered?
4. What are the potential consequences of closing the operating room dedicated to emergencies?

We use the most common performance indicators used in practice and in the literature such as utilization, over- and undertime, waiting time of patients, the number of treated patients, and the number of deferred surgeries to evaluate these questions. The results provide a quantitative basis for future operating room planning and scheduling. After the conclusion of the project, insights generated from our study served to calibrate the newly implemented planning software.

### Patient grouping

The medical condition of patients has important implications for the management of their surgery. It determines whether there is enough time to include them in a weekly surgery plan or they need to be inserted into an existing plan, hence disrupting an existing plan. Additionally, it influences the expected duration of their surgery procedure. After extensive data analysis and feedback from the collaborating OR-manager, the following distinction of patient acuity levels in four groups has proved useful:

1. Elective patients (surgery can be scheduled well ahead of time)
2. Semi-urgent patients (surgery within 24 h after arrival)
3. Very-urgent patients (surgery within 6 h after arrival)
4. Emergency patients (surgery a soon as possible after arrival)

The condition of elective patients permits enough time to schedule their surgery well ahead of time, so they become part of the weekly surgery scheduling process.

Our practice partner had previously used a single “urgent” category for patients that do not qualify as emergencies but should receive treatment soon after their arrival. However, some of these urgent patients need to undergo surgery within 6 h, while others only require treatment within the next 24 h. This leads to two different policies applied to urgent patients in the current day-to-day process. To model both policies in our simulation, we separate urgent patients into semi-urgent and very-urgent patients. Whenever we mention urgent patients, we refer to both semi- and very-urgent patients as is current practice at the hospital.

### Surgery scheduling at the university hospital Augsburg

The central operating theater consists of 18 operating rooms. Out of these, one room is dedicated to emergency patients only. In this room, surgeons and anesthesiologists are on staff day and night, and supporting personnel (e.g., circulating nurses) are available on short notice in case of an emergency. Also, two of the operating rooms run throughout the night to handle very-urgent cases. There are 13 rooms available for the induction of anesthesia.

**Fig. 1:**
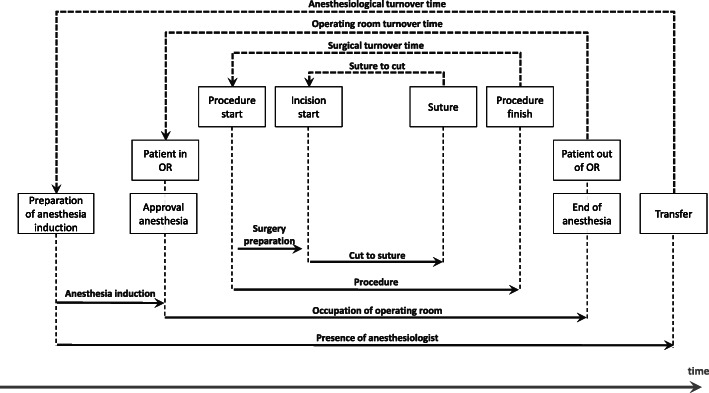
Master surgery schedule

The master surgery schedule (MSS) predetermines the daily assignments of each operating room to medical departments. An MSS holds for every week until the next revision takes place. We illustrate the MSS for the considered period in Fig. 1. The assignment of “E” indicates that the room is reserved for emergencies and no treatment of elective patients occurs there. The legend of Fig. 1 lists the abbreviations of the different department names used in the remainder of this paper.

The departments set up preliminary schedules for their respective patients independently of each other, based on the current allocation of the MSS. These schedules, in general, follow the logic to fill regular hours with semi-urgent and elective patients. These schedules are available no later than the day before surgery.

An OR-coordinator is responsible for coordinating the individual schedules of the departments and for disruption management. Emergency patients are assigned to the emergency operating room or the next available operating room. If the emergency patient is assigned to any room other than the emergency operating room, the remaining patients on the room’s schedule are postponed accordingly. Should this cause elective patients to be moved outside of the operating hours, they are deferred to the next day and appended to the elective program of the respective department.

In practice, options such as the reallocation of operating rooms, the extension of opening times, and postponing surgeries are available to manage short-term disruptions in the surgical program.

### Surgery process description

**Fig. 2:**
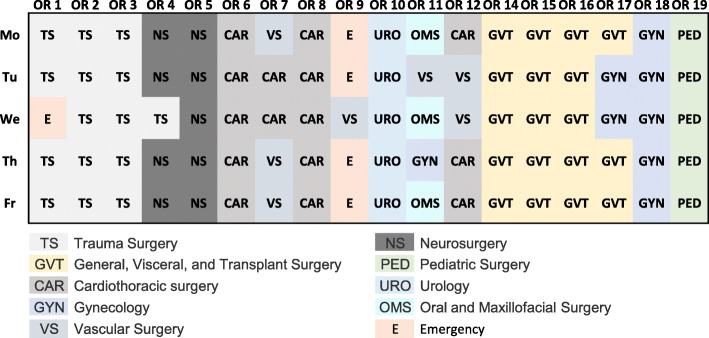
Surgical sub-processes

A single surgery consists of several sub-processes. Figure 2 illustrates the most common process flow and the timestamps collected by the information technology system at University Hospital Augsburg. For some types of surgeries, this flow can partially deviate, e.g., for procedures performed under local anesthesia. Patients wait in the holding area until their first contact with the anesthesiologist. The first injection of anesthetics marks the start of the induction. After the anesthesia team completes the induction of anesthesia, they move the patient to the operating room. The duration between the start and the end of the procedure coincides with the presence of the surgeon. The time from the first cut to suture is widely used in productivity benchmarks. After the surgical follow-up, the anesthesiologist moves the patient out of the operating room to either the post-anesthesia care unit (PACU) or the intensive care unit (ICU), where a handover takes place. The anesthesiologist monitors the patient continuously from the induction until the transfer to a downstream unit. As long as no parallel induction of anesthesia - one of the proposed managerial changes - is performed, the “presence of anesthesiologist” duration determines how much time a single patient occupies a surgery team and therefore the OR resource (even though not physically for the entire time) until the next patient can be treated. In a setting where parallel inductions are used, this occupancy duration shrinks to only the “occupation of operating room” duration.

### Investigated research questions linked to management policy options

Each of the investigated management policy options is compared to current practice, modeled as the Base Case. In the Base Case, patient sequencing follows a First In First Out (FIFO) logic, semi-urgent patients are not scheduled at night time, operating rooms are open during regular hours, and parallel induction of anesthesia is not utilized. To address the most important considered managerial decisions, we conducted the following analyses:

#### Partial extension of opening hours

Deferring treatments to the next day can trigger planning problems like rescheduling and further postponement of procedures. The introduction of extended opening hours for a selection of operating rooms serves as a buffer to react to schedule disruptions, as patients are only scheduled over the regular opening hours of an OR with extended opening hours. The additional capacity is used to finish delayed surgeries or take on pending treatments from other operating rooms. This reduces the need for deferrals and prevents planning difficulties on the next day. However, regularly offering additional opening hours is an expensive endeavor, so we quantify the improvements to patient flow caused by the extension of opening hours.

#### Overlapping induction of anesthesia

A team consisting of an anesthesiologist and an anesthesia nurse is responsible for each anesthesia. In the Base Case, the induction of anesthesia of a patient starts once the surgical procedure of the previous patient is finished. Since the anesthesia team also observes the narcosis of the patient during the surgery procedure, it is engaged over the complete surgery duration. During the induction of anesthesia, the operating room and the surgery team remain idle. This implies that long induction times cause a lower utilization of the OR. Overlapping induction of anesthesia provides a way to decrease this OR idle time. Separate rooms allow the anesthesia team to start the induction of anesthesia of the following patient before the previous patient leaves the operating room if sufficient anesthesia teams are available. Since the first patient in a session has no predecessor, his induction is not overlapping.

The relocation of inductions to other rooms and the execution parallel to surgical procedures in the operating rooms effectively increases OR surgery capacity. When this additional capacity exceeds a certain threshold, additional patients from the waiting list can be scheduled, which increases the number of treated patients in the entire operating theatre.

#### Patient sequencing: first in first out (FIFO) vs. shortest first (SF) vs. longest first (LF)

Patient scheduling at the University Hospital Augsburg utilizes a schedule waiting list consisting of three patient categories that are sorted by priority. Patients within each category are ordered by a FIFO logic; they are not sorted by their expected surgery durations. The FIFO sequencing strategy, therefore, creates a schedule that is random in terms of expected surgery durations. Under a Shortest (Longest) First policy, the same patient selection logic is applied, but the patients on the resulting schedule are eventually sorted by expected surgery durations in ascending (descending) order, i.e. the patients on the schedules remain the same, only their sequence changes.

#### Closing the dedicated emergency operating room

During the day, one operating room is staffed and on stand-by for possible incoming emergency and very-urgent patients in current practice. The relatively low number of emergency and very-urgent arrivals, however, causes a low utilization of the ORs. We investigate how average waiting times, patient deferrals, and expected over- and undertime change when the OR is closed. Our goal is to assess the risks associated with this revised policy, which would offer the immediate benefit of reducing operating costs in the operating theater.

### Key performance indicators

We measure several performance indicators to track the influence of scenario changes on the simulated system. The most important indicators are: Number of treated patients, utilization of the operating rooms, overtime and undertime in the operating rooms, waiting time of patients, and the number of deferred patients, which we define as patients whose surgeries are performed on a later day than originally planned.

Utilization rates of the operating theater are reported for regular time (08:30 am to 03:45 pm) and extended opening times (03:45 pm to 08:00 pm). They can be further separated into system utilization - including anesthesia, surgery, and cleaning times - and room utilization, which considers surgery and cleaning times only. This is especially important when analyzing the effects of parallel anesthesia inductions.

Overtime / undertime measures the number of minutes that operating rooms run longer / shorter than the planned opening hours according to the MSS. They serve as a proxy for the expected deviation of staff work time from contractually agreed levels, assuming that staff scheduling is initially done according to said levels.

Patient waiting times can be reported for each patient acuity type (elective, semi-urgent, very-urgent, and emergency). They are a measure of the delay between the planned start and the actual start of a patient’s surgery. When elective patients are deferred to the next day because of delays in the schedule, their waiting time will not begin at the originally planned start, but instead at the updated planned start on the next day. The number of patients that need to be deferred from 1 day to the next is also an important performance measure. Thus, our definition of patient waiting times measures intraday delays in the schedule, while the number of deferrals is a measurement of interday rescheduling.

### Available hospital data

Twelve thousand nine hundred forty-six surgical procedures were performed in the central operating theater between October 2015 and July 2016. Nine different departments performed more than 250 procedures during the time period, making them an integral part of the central operating theater planning. The remaining surgical departments used the operating theater very sparsely and irregularly and were excluded from our study. The nine considered departments performed 2.9 surgeries per day per operating room on average, resulting in 345 surgeries per week. Our data analysis was performed using the R Project for Statistical Computing (R) software.

### Patient grouping by acuity

**Fig. 3:**
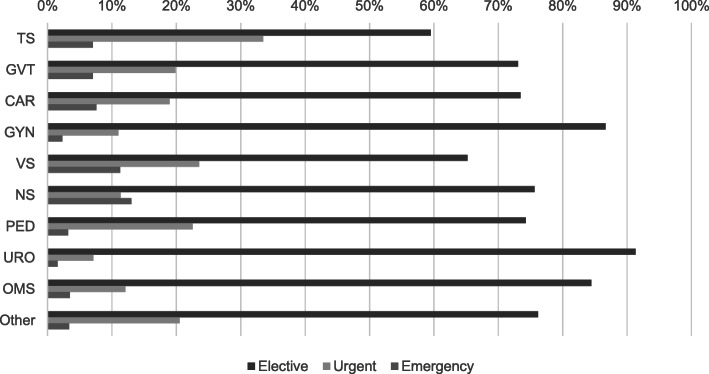
Urgency categories by department

At the University Hospital Augsburg, emergency and urgent patients form a considerable proportion of the patient population. Thus, daily disruptions to the planned schedule are common. Elective patients account for 72% of all patients, 21% are urgent (note that the hospital did not use the subgroups “semi-urgent” and “very-urgent” before our study), and 7% are emergency patients. It is therefore important to consider their impact on the performance of any investigated OR management policy. Figure 3 reveals the diversity of the different departments for urgency categories. A high share of urgent patients, for example, occurs within the department for Trauma Surgery. Gynecology and Urology are the departments with the highest proportion of elective patients with over 85% of all patients. About half of the urgent patients are very-urgent and the other half semi-urgent. This assessment resembles the experience of the OR-manager.

### Surgery durations and patient classification

**Fig. 4:**
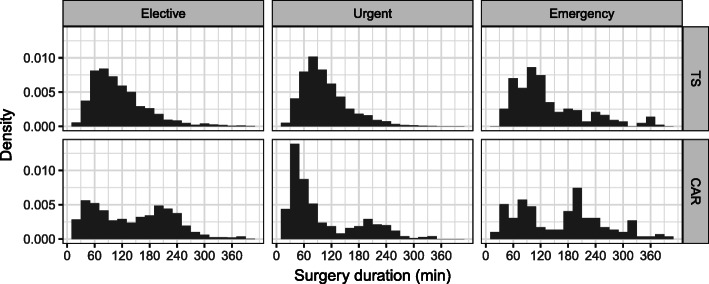
Distributions of surgery durations

The differences in surgery durations between the specialties and urgency categories must be considered, because surgery durations have a direct impact on performance measures such as utilization and overtime. Histograms for surgery durations of the specialties TS and CAR are depicted in Fig. 4 to illustrate exemplary structural peculiarities. The surgery durations in CAR, for example, can be modeled using a bimodal distribution, whereas the distribution of the surgery durations at TS resembles a log-normal distribution. The average surgery duration, as well as the standard deviation of the surgery duration, is notably higher for emergency patients than for elective patients in both specialties. Urgent CAR patients are characterized by their short surgery duration compared to the other urgency categories.

Anesthesia times are relevant for patient scheduling unless parallel induction of anesthesia is performed. The empirical distributions of anesthesia times are further clustered according to the surgery duration. Hereby, we assume that the surgery planner only has an approximate forecast regarding the surgery duration. The times used for scheduling elective patients are displayed in Table 1.

**Table 1:** Average Presence of Anesthesiologist Times of Elective Patients in Minutes

Specialty	Short	Medium	Long
GVT	74	166	371
VS	46	135	334
GYN	44	101	322
CAR	63	204	324
PED	40	97	256
OMS	55	137	307
NS	81	194	406
TS	63	134	288
URO	94	210	375

### Emergency and urgent patient arrivals

Urgent patient arrival rates vary across different departments since urgent patients are assigned to operating rooms of their respective specialty, if possible. Table 2 provides an overview of the different average urgent patient arrivals between 6 am and 10 pm on weekdays for each department. Urgent patients mainly arrive during the day. Only a total of 0.48 urgent patients are recorded on average per night.

**Table 2:** Daytime Arrival Rates of Urgent Patients per Weekday

Specialty	Mean	SD
TS	4.7	2.3
GVT	2.4	1.4
CAR	1.8	1.5
GYN	0.6	0.8
VS	1.3	1.2
NS	0.5	0.7
PED	1.0	1.0
URO	0.1	0.4
OMS	0.2	0.5
**Total**	**12.6**	

Emergencies are assigned to the next available operating room independent of the specialty this room is assigned to. Thus, the arrival rates of emergency patients only differ with respect to the time of day. Between 6 am and 10 pm, 2.8 emergencies arrive on average with a standard deviation of 1.9, and, during the night, an average of 0.8 emergencies arrive with a standard deviation of 1.1.

## Results

Each of the following analyses targets one of the aforementioned research questions. The simulation was programmed and implemented in AnyLogic. The performance measures and confidence intervals were analyzed using Excel.

### Extended operating hours

**Fig. 5:**
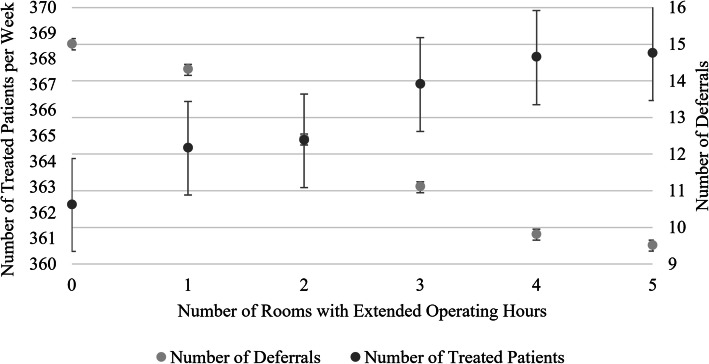
Trade-off between treatments and deferrals

Our results indicate that the number of additional patients treated in a week increases slightly per room with each additional operating hour. These additional patients are mostly patients who would otherwise have to be deferred to the next day, as the reduction in the number of deferred elective surgeries shows. (see Fig. 5; in all figures, we depict the mean and the 95% confidence interval – if the intervals do not overlap, the results are statistically significantly different from one another at the 0.05 confidence level). While the increase in the number of treated patients is only statistically significant when extending operating hours by more than three, the number of deferrals shows very little fluctuation, and the reduction is statistically significant with each additional opening hour.

**Fig. 6:**
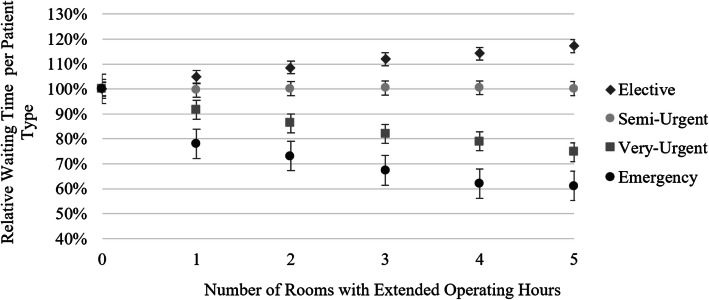
Impact of extended hours on waiting time

Interestingly, there is a striking decrease in the waiting time for emergency and very-urgent patients as operating room hours increase. Semi-urgent and elective surgery patients, on the other hand, have longer intraday waiting times as operating rooms are opened longer, on average (see Fig. 6).

### Parallel induction of anesthesia

**Fig. 7:**
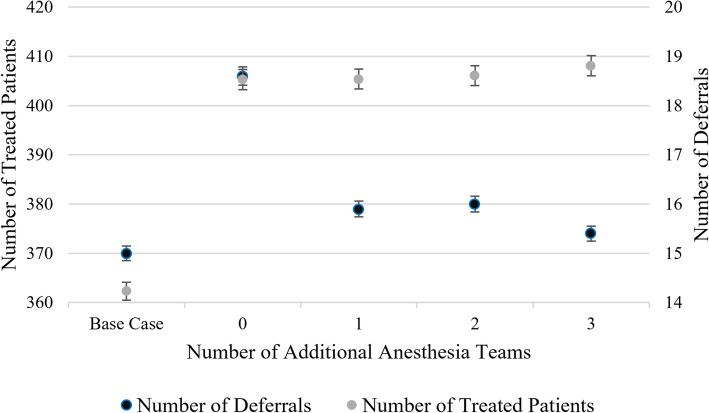
Effects of parallel induction on patients

Introducing the policy of conducting parallel induction of anesthesia leads to a second decision of how many additional anesthesia teams, consisting of an anesthesiologist and a nurse anesthesiologist, to staff. We compare the Base Case, which represents the current practice of performing sequential anesthesia inductions, with settings in which parallel induction is implemented and zero to three additional anesthesia teams are employed. Our results indicate that parallel induction leads to approximately 10% more treated patients thanks to a higher number of elective patients that fit into the schedule (see Fig. 7). This gain comes at the cost of a significantly increased number of deferrals, however, which can be alleviated with at least one additional anesthesia team. Employing more than one additional anesthesia team yields no or hardly any improvements concerning the number of treated patients or deferrals.

**Fig. 8:**
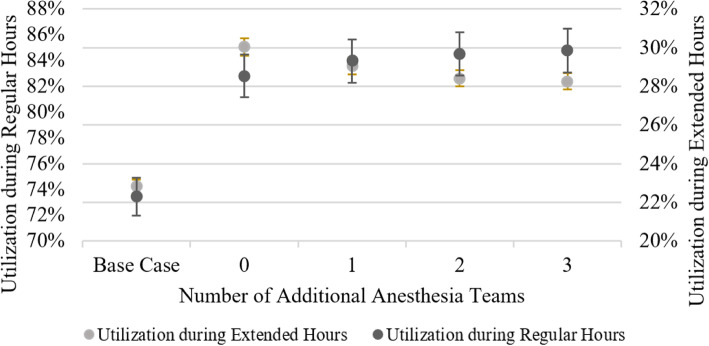
Impact of parallel induction on OR utilization

Increasing the number of anesthesia teams does help increase OR utilization during regular hours while simultaneously decreasing OR utilization in the extended hours (see Fig. 8), hence reducing disruptions in the schedule that result in longer patient waiting times.

### Sequencing patients

While the Base Case assumes a random daily sequence of scheduled patients regarding their expected surgery duration, we investigate the impact of different sequencing policies for the scheduling of elective and semi-urgent patients. Three different sequencing policies are considered:

1. First In First Out (FIFO): The sequencing of expected surgery durations follows no explicit sorting policy. Concerning surgery durations, the daily operating sequence is random.
2. Longest first (LF): The longer the expected surgery duration, the earlier in the day the patient is scheduled.
3. Shortest first (SF): The shorter the expected procedure duration, the earlier in the day the patient is scheduled.

The results of different sequencing policies are depicted in Table 3, where we report the 95% confidence intervals for each performance measure under each sequencing policy. Note that results are statistically significantly different from one another at a 0.05 level of significance if the reported intervals do not overlap. SF increases the number of surgeries per week compared to the currently employed FIFO sequence. LF yields the lowest number of weekly cases. The same level of performance is mirrored concerning the number of deferred patients. Operating room utilization, on the other hand, is highest under an LF policy and lowest under an SF policy. Overtime can be expected to be highest with an SF policy and lowest with an LF policy. Emergency patients wait the shortest with the FIFO policy, but the differences are within one and a half minutes. Anesthesia turnover time is also lowest when a FIFO policy is employed.

**Table 3:** Results of Different Sequencing Policies

Sequence policy	Treated cases per week	Patient deferrals per week	Room utilization	Overtime per room per day	Waiting time of emergencies	Anesthesia turnover time
FIFO	[361.1, 363.5]	[14.4, 15.6]	[71.8, 74.0%]	[25.5, 27.1]	[4.1, 4.9]	[12.5, 13.1]
LF	[356.7, 358.9]	[21.1, 22.9]	[72.5, 75.1%]	[24.2, 25.4]	[5.2,5.6]	[13.9, 14.7]
SF	[365.6, 368.6]	[8.8, 10]	[69.9, 72.1%]	[33.7, 34.7]	[5.0,5.6]	[15.0, 16.0]

### Closing the dedicated emergency OR

Not surprisingly, shutting down the dedicated emergency operating room results in fewer treated cases per week (see Table 4). Likewise, an increase in the waiting time of emergency patients can be expected. More interesting is the very small increase in the waiting time of elective patients by a mere couple of minutes. Also, the number of patient deferrals increases only by about 2 patients per week. Overtime can be expected to increase by a few minutes when no operating room is reserved for emergencies, but there is a noticeable resulting reduction in undertime, i.e. idle time at the end of the operating hours.

**Table 4:** Closing the Dedicated Emergency Room, Time Units in Minutes

Emergency room policy	Treated cases per week	Waiting time of emergencies	Waiting time of electives	Patient deferrals per week	Overtime per room per day	Undertime per room per day
Dedicated emergency OR	[361.1, 363.5]	[4.1, 4.9]	[29.6, 31.8]	[14.0, 16.0]	[25.6, 27.0]	[27.7, 29.1]
No dedicated emergency OR	[352.2, 356.4]	[21.3, 24.1]	[32.6, 33.6]	[16.5, 18.3]	[29.0, 30.2]	[22.9, 24.5]

## Discussion

While longer opening hours of operating rooms could allow the scheduling of additional surgeries, University Hospital Augsburg was foremost interested in how added room capacity might help reduce negative effects for a given schedule. Therefore, we treat the added opening hours (up until 8:00 pm) for each operating room as buffer capacity that may be used to perform elective surgeries at the end of the day that would have otherwise had to be deferred until the next day, but no elective surgeries are actively planned during these extended hours. This results in a rising number of semi-urgent patients that can be treated on the day of their arrival. Of course, the additional OR capacity may also be used to schedule additional patients rather than simply working as buffer capacity at the end of the day, providing more financial incentives through increased revenue to offset the increased personnel cost. The fact that elective patients are deferred less frequently and can therefore be treated on the day of their scheduled surgery more often contributes significantly to their increased intraday waiting time. In this case, they are moved to the end-of-day buffer time more frequently, hence incurring waiting time but foregoing very undesirable deferrals. If they were deferred to the next day, they would not encounter any waiting during the day of the originally planned surgery per our definition. In practice, a reasonable intraday waiting time will be preferred by patients and hospital management over a deferral to the next day.

Parallel induction means that surgeries can be scheduled according to their expected occupancy of the operating room rather than their required presence of anesthesiologist time, resulting in a tighter schedule. It is important to note that even in the case of parallel induction with zero additional anesthesia teams, the team that staffs the operating room reserved for emergencies may serve as a backup for elective surgeries when no emergency patient is present. Thus, some parallel induction may always be employed in our setting, even if no extra personnel is scheduled to be available. However, if there exists no anesthesia team dedicated to performing parallel inductions, it is not advisable to adjust the operating schedules by eliminating the planned anesthesia time from the expected surgery duration. It may prove useful to utilize parallel induction of anesthesia especially for long surgeries in combination with a sensible policy that sequences surgeries according to their expected duration. Marjamaa et al. [16] discuss the cost efficiency of parallel induction. In their simulation setting, parallel anesthesia outperforms the traditional sequential setting concerning cost-efficiency in any organizational layout. Basto et al. [17] find, as we do, that parallel induction reduces intra-operative times and therefore allows for more surgeries to be performed. However, our study incorporates the fact that parallel induction of anesthesia allows for tighter surgery schedules to maximize the cut-to-suture time utilization of the operating rooms. Therefore, we can show the trade-off between higher utilization as well as throughput and reduced planning stability, which can only partially be mitigated by employing additional anesthesia teams.

Interpreting the results of the analysis of different sequencing policies correctly might not be straightforward. Under an SF policy, longer than expected surgery durations or unforeseen emergency surgeries in an operating room will often cause only a single patient, the one who is treated last because he has the longest expected surgery duration of the day, to be deferred to the next day. In contrast, under an LF policy, a similar delay might result in the deferral of several (short) surgeries. Hence the differences in treated cases and patient deferrals per week. Associated with this observation is the remarkable difference in overtime. As delays in the schedule might result in the deferral of the last patient to the next day under the longest first policy (because the surgery cannot be performed even during extended opening hours), the shortest first policy often allows starting the last patient of the day within the opening hours at the expense of overtime. The average waiting time of emergency patients is lowest when using a FIFO sequencing policy, which is explained by the generation of more break-in-moments [18], which provides more opportunities to slot emergency surgeries into the schedule. Overall, the FIFO sequence yields a preferable set of results for our practice partner. For each of the other two sequencing policies, improvements in one or two performance measures are offset by stronger deteriorations of other performance measures.

When the dedicated emergency room is shut down completely and not used for the regular program of any specialty, our results illustrate the degrading performance of the entire operating theater. The most remarkable change occurs in the emergency patient waiting time. Its mean value increases significantly from 4.5 to 22.7 min. However, an average waiting time of 22 min itself may be acceptable, as in the majority of cases the OR-manager knows of incoming emergencies around 30 min before they eventually enter the operating room. This will usually provide enough time to find an OR where a surgery is coming to an end or no surgery is scheduled. But there will still be an increased risk of having no OR capacity available when the emergency patient has arrived and the surgery should start. With this information, management can gain a better understanding of the trade-off between cost savings and performance deterioration. Wullink et al. [19] present a simulation study that shows how closing a dedicated emergency OR while simultaneously reserving capacity for emergencies in the elective ORs yields overall better results concerning OR utilization and emergency patient waiting time. However, they do not report results regarding the number of elective surgeries than can be performed. While our results concerning emergency patients match their findings, we identify the reduced number of performed elective surgeries as a shortcoming of their proposed policy. Ultimately, our practice partner decided that the dedicated emergency OR should remain in place for the time being.

There are of course limitations to our study that should not go unmentioned. First of all, our simulation study overestimates the number of performed surgeries in the Base Case, on average, by about 5%. The main reason for this is the fact that, in reality, there are various uncommon but present interruptions in operating theaters. These include the absence of hospital personnel, issues with intrahospital patient transportation, patients who do not show up for elective surgeries on time or with an empty stomach, or missing paperwork. Furthermore, our study addresses operating theater management in a large maximum care provider hospital with multiple medical disciplines, which may not be comparable to other types of hospitals such as specialized clinics for a small number of standardized procedures. Finally, medical departments at the University Hospital Augsburg dictate surgery schedules, not the surgeons. This provides more flexibility in our setting that might be present when surgeons are tied to their patients, as is often the case in hospitals in the United States, for example.

Further research could consider congestion levels and anticipated available capacity in downstream units, a relevant reason for cancellations of surgeries [20, 21] that we did not address in this study. Additionally, more advanced patient sequencing techniques that have been presented and evaluated in the scheduling literature [22–24] could be implemented in the simulation model. More advanced forecasting techniques with regards to the distribution of surgery durations for certain patient types could be employed as well. Especially machine learning techniques could provide opportunities to map patient characteristics to expected surgery durations when large-scale data is available data.

## Conclusions

The study presented in this paper is part of a close collaboration between the authors and the University Hospital Augsburg. Based on deliberations to introduce a multitude of changes to current management practices in their large operating theater, we developed a detailed simulation of both the scheduling process and the online management decisions. A large dataset with detailed information on each of over 10 thousand surgeries performed between October 2015 and July 2016 serves as input for our analyses. Our approach allows us to capture various intra- and inter-day effects of changes to the scheduling policies and the handling of schedule disruptions to analyze their potential benefits. Furthermore, we incorporate detailed disruption management, e.g. preponements and operating room reassignments, as well as realistic rescheduling of deferred surgeries, enabling us to derive insights into resource idle times, cancellation rates and waiting times of individual patients. The close interaction between the operating room management and our team allowed us to achieve such a high level of detail in the simulation model. Thus, we can reliably evaluate potential changes in management policies – arguably better than (more abstract) analytical models would have allowed us to. Typically, the proposed managerial changes lead to trade-offs between the number of performed surgeries and quality-related performance measures such as waiting time, overtime, or surgery deferrals. While the existence of such trade-offs is mostly unsurprising, managers need to understand the magnitude of the expected changes in performance measures.

Lessons learned for our practice partner comprise an adaption and an increase in the number of timestamps that are collected during each surgery. Moreover, the category of urgent patients was refined to separate semi-urgent from very-urgent patients following our project. Finally, the planned surgery duration used in scheduling has changed from merely representing the average historical duration to be the result of a dynamic forecast system, so that new surgery data influence the forecast of future procedures.

## Supplementary Information

**Additional file 1.**

## Data Availability

The datasets used and/or analyzed during the current study are available from the corresponding author on reasonable request if the University Hospital Augsburg authorizes the recipient to receive the data.

## Abbreviations

CAR: Cardiothoracic surgery
E: Emergency
FIFO: First in first out
GVT: General, visceral, and transplant surgery
GYN: Gynecology
ICU: Intensive care unit
LF: Longest first
MSS: Master surgery schedule
NS: Neurosurgery
OMS: Oral and maxillofacial surgery
OR: Operating room
PACU: Post-anesthesia care unit
PED: Pediatric surgery
SF: Shortest first
TS: Trauma surgery
URO: Urology
VS: Vascular surgery
