# Supplementary material for: Simulation-based evaluation of operating room management policies
Source: BMC Health Serv Res. 2021 Mar 24;21:271. doi: 10.1186/s12913-021-06234-5 (PMC7992985; doi:10.1186/s12913-021-06234-5)
Supplement: Supplementary file 1 — Additional file 1. [file 12913_2021_6234_MOESM1_ESM.docx]

# Technical Appendix

# Simulation design

The simulation is set up to model the investigated management policies and their impact on key performance indicators on the tactical and operational levels. It incorporates decisions on weekly patient scheduling, operating room capacity, disruption management, and patient flow in the main operating theater of the University Hospital Augsburg. The required personnel capacity directly links to the operating room opening hours and is only tracked as a resulting performance measure. Hence, we assume that personnel is present whenever a room is open, as our focus does not lie on personnel planning. The simulation covers a week from Monday to Friday, because only emergency surgeries are performed and no elective patients are scheduled on the weekend. The results are based on 1,000 independently simulated weeks.

## *Scheduling of Patients in the Simulation*

Each medical specialty schedules patients from the waiting list independently from the other departments. Hence, each specialty has its own waiting list of patients. The scheduling typically happens on the preceding afternoon. In our model, the number of randomly generated elective patients on the waiting list large enough to fill an entire week. These patients are ordered in a FIFO sequence, so that the simulation cannot freely pick any order of patients to optimize the schedule with only utilization maximization in mind. This assumption is reasonable because every department at the University Hospital Augsburg was able to fill all available surgery times with patients during the time of the project, and patients wait longer than one month on average for an elective procedure in European hospitals [1].

The scheduling of a patient entails both the starting time of the procedure and the duration for which the OR is reserved for the surgery. Since every department at the University Hospital Augsburg autonomously schedules their patients, there exist no strict scheduling policies. Unfortunately, we were unable to obtain past surgery schedules. Thus, we had no means to measure the accuracy of the scheduled surgery durations. The leading manager of the operating theater confirmed that it is common practice to assign time slots according to the expected duration of a procedure at the University Hospital Augsburg. We create three patient clusters from historical data on patients from each specialty in our model to mimic the current scheduling behavior. The cluster with the short durations contains every procedure that took up to the 30% percentile of the historically observed durations. The medium duration cluster includes surgeries with durations between the 30% and the 90% percentile. All procedures longer than the 90% percentile form the long duration cluster.

Every elective patient on the waiting list is part of one of the three clusters within their respective medical specialty. The probability that a patient is part of the short/medium/long cluster is 30%/60%/10%, respectively. The within-cluster expected anesthesia and operating procedure durations, plus an average post-surgical cleaning time, then determine the scheduled duration of their surgery. The resulting scheduling logic under the current scheduling policy, as well as an alternative scheduling policy with parallel induction of anesthesia, are depicted in Figure A1.


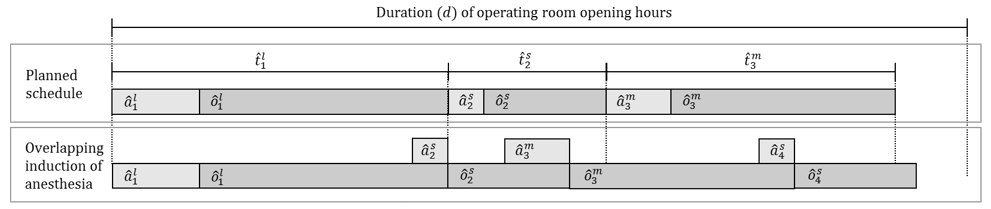


Figure A1

In addition to elective patients, semi-urgent patients who need to be treated within 24 hours are usually treated on the day following their arrival. Therefore, these patients move to the top of the waiting list and are the first to be scheduled (see Figure A2). Their planned surgery duration is determined similarly to the previously described method for elective patients. Elective patients, who had to be deferred on the previous day because of unforeseen delays, complete the waiting list. These patients have a lower priority than semi-urgent cases, but they are scheduled ahead of new elective patients in the program.

The sequence of decision making in the scheduling process follows the flow chart depicted in Figure A2. Each medical specialty that has at least one room assigned to them follows the same logic. As long as they have enough capacity available in (one of) their room(s), they assign OR time to patients on the waiting list from top to bottom. Towards the end of the day, when OR capacity is insufficient to fit the next patient on the waiting list, they go down the waiting list and look for patients with suitable expected durations to fill up the remaining time. Once not even short surgeries fit into the schedule of an OR, scheduling for the particular OR is finished for the day.


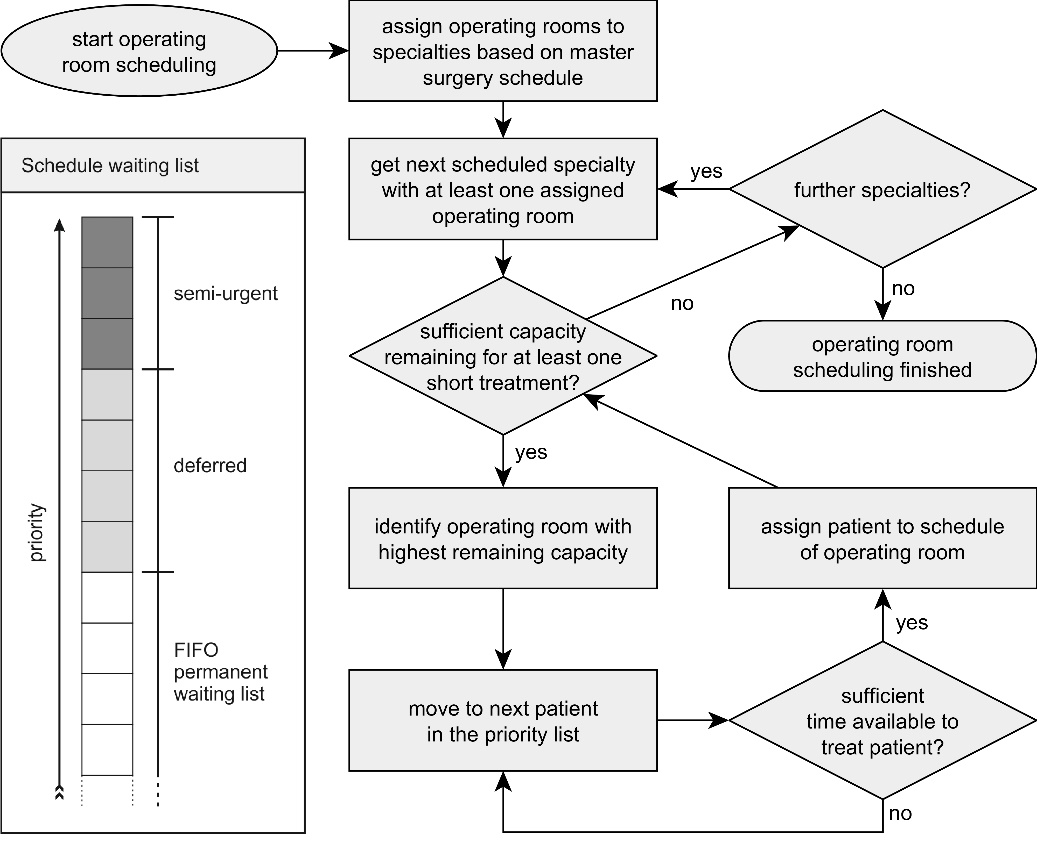


Figure A2

## *Operating Room Management in the Simulation*

An operating theater schedule is often disrupted by stochastic incidents like complications during surgery or incoming very-urgent or emergency cases. In these cases, efficient disruption management needs to handle the situation. Online management of the operating theater is a complicated task. In our simulation, we model the processes in as much detail as possible and necessary.

Emergencies are usually treated in the dedicated emergency operating room. However, if this room is occupied, they are transferred to the next available room to ensure the quickest possible treatment. If no room is available at the time of their arrival, they wait for a room to become available. Very-urgent patients are treated differently. Upon their arrival, they are assigned to the next idle room of their respective specialty. If there is no room available in the next six hours or the treatment of the patient would lead to more than three hours of overtime, the patient is brought to the dedicated emergency room. If this room is occupied as well, the patient is transferred back to a queue in front of the room of their specialty. An overview of the workflow of a single room is depicted in Figure A3.


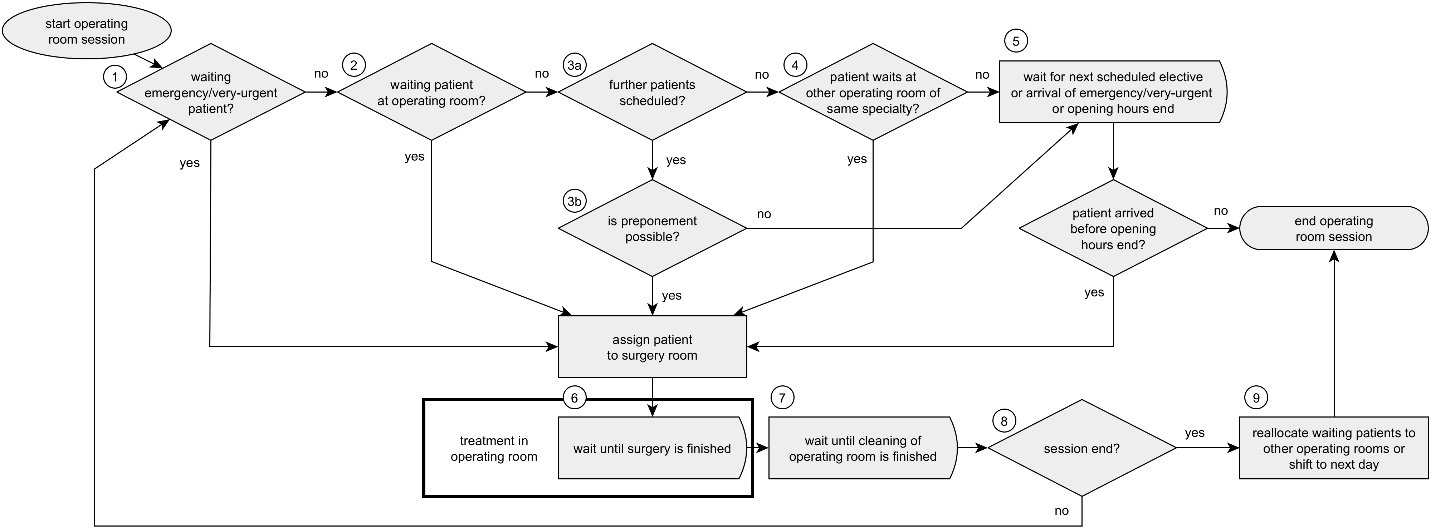


Figure A3

Assignments of potentially waiting emergency or very-urgent cases to the operating room are performed first (1). They have priority over the patients from the schedule waiting list. All remaining scheduled surgeries in the room and the regular program of the room is then postponed. If no emergency or very-urgent patients are waiting, a check is performed if there are regularly scheduled patients already waiting for surgery (2). If that is the case, the patient is sent to the operating room immediately. Otherwise, we check if there is another patient scheduled in the room (3a). If another patient is scheduled and there are at least 30 minutes until this patient’s scheduled surgery, there is a 90% likelihood that a preponement is possible (3b), which would mean that the patient is sent to the room right away. If no further patient is scheduled, we try to acquire a waiting patient from another room of the same specialty (4). If this is not possible, the room stays idle. We then wait until the next patient arrives (5), similar to when a patient scheduled for surgery at a later date could not be preponed. An emergency or a very-urgent patient may arrive before the next scheduled patient in (5). If neither happens and time runs out, the operating room session ends for the day. Once a patient is sent to the operating room, the treatment begins (6). The actual surgery duration for each patient is randomly drawn from the continuous empirical distribution of the respective acuity group in the specialty. While every schedule consists of a sequence of expected surgery durations$\hat{p}_{n}$, the realized session consisting of consecutive randomly drawn durations $p_{n}$ will typically deviate from the schedule (see A4). Once a surgery ends earlier and a preponement of the subsequent procedure is not viable, idle time $s_{n}$ occurs. Conversely, surgeries that take longer than expected cause a delay to the start time of subsequent procedures ($w_{n})$. Under this condition, the application of specific scheduling policies can help to reduce idle time $s_{n}$, waiting time $w_{n}$ and overtime $l$. After each procedure, the room has to be cleaned. The duration for the $i$-th cleaning in room $r$ ($d_{ir}^{c}$) is modelled in minutes as $d_{ir}^{c}\sim triangular\left( 5, 7.5, 10 \right)$, which is, according to the OR-manager, an appropriate assumption, as we classify surgeries by the performing medical departments and expected duration, but not the exact procedure. The latter would allow accounting for variances in cleanup times depending on the procedure (e.g. moving special equipment), but it would result in a multitude of drawbacks for our study, because it would reduce the number of surgeries per category in our data set drastically, despite a large amount of available data. After the surgery and cleaning (7), we check if the room is still open for further procedures [8]. Should this be the case, we loop back to (1), otherwise any potential remaining waiting patients are reallocated to other rooms of the same specialty that are still open or postponed to the next day (9).


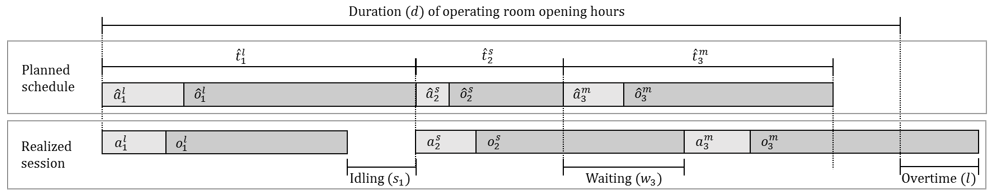


Figure A4

Figure A1: Scheduling logic

Figure A1 Legend:


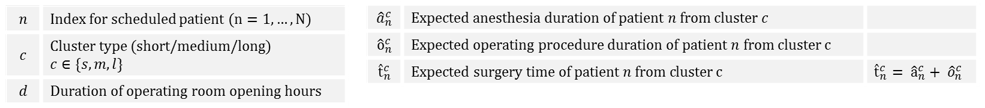


Figure A2: Scheduling logic

Figure A3: Operating room process

Figure A4: Definitions of idling, waiting, and overtime

Figure A4 Legend:


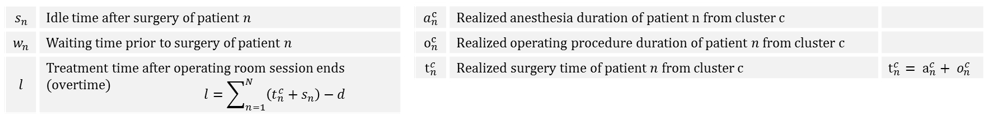


**Reference**

1. Viberg N, Forsberg BC, Borowitz M, Molin R. International comparisons of waiting times in health care–limitations and prospects. Health Pol. 2013; 112(1):53–61.
